# The mental representation of the human gait in young and older adults

**DOI:** 10.3389/fpsyg.2015.00943

**Published:** 2015-07-14

**Authors:** Tino Stöckel, Robert Jacksteit, Martin Behrens, Ralf Skripitz, Rainer Bader, Anett Mau-Moeller

**Affiliations:** ^1^Department of Sport Science, University of RostockRostock, Germany; ^2^Human Motor Control Laboratory, Psychology, School of Medicine, University of TasmaniaHobart, TAS, Australia; ^3^Department of Orthopaedics, University Medicine RostockRostock, Germany

**Keywords:** structural dimensional analysis of mental representation (SDA-M), long-term memory, normal and pathological gait, aging

## Abstract

The link between mental representation (MREP) structures and motor performance has been evidenced for a great variety of movement skills, but not for the human gait. Therefore the present study sought to investigate the cognitive memory structures underlying the human gait in young and older adults. In a first experiment, gait parameters at comfortable gait speed (OptoGait) were compared with gait-specific MREPs (structural dimensional analysis of MREP; SDA-M) in 36 young adults. Participants were divided into a slow- and fast-walking group. The proven relationship between gait speed and executive functions such as working memory led to the hypothesis that gait pattern and MREP differ between slow- and fast-walking adults. In a second experiment, gait performance and MREPs were compared between 24 young (27.9 years) and 24 elderly (60.1 years) participants. As age-related declines in gait performance occur from the seventh decade of life onward, we hypothesized that gait parameters would not be affected until the age of 60 years accompanied by unchanged MREP. Data of experiment one revealed that gait parameters and MREPs differed significantly between slow and fast walkers. Notably, eleven previously incurred musculoskeletal injuries were documented for the slow walkers but only two injuries and one disorder for fast walkers. Experiment two revealed no age-related differences in gait parameters or MREPs between healthy young and older adults. In conclusion, the differences in gait parameters associated with lower comfortable gait speeds are reflected by differences in MREPs, whereby SDA-M data indicate that the single limb support phase may serve as a critical functional period. These differences probably resulted from previously incurred musculoskeletal injuries. Our data further indicate that the human gait and its MREP are stable until the age of 60. SDA-M may be considered as a valuable clinical tool for diagnosis of gait abnormalities and monitoring of therapeutic effectiveness.

## Introduction

It is widely accepted that skilled motor performance draws on general and task-specific mental representations (MREPs) that are linked to perceptual effects and correspond to functional structures of movement kinematics (cf. Bernstein, 1967; Hommel et al., 2001; Schack and Ritter, 2009; Land et al., 2013). MREPs are developed and established with increasing amounts of practice and are regarded to be crucial for the organization and control of actions. The link between well-established MREP structures and motor performance has been evidenced for a great variety of movement skills (see Land et al., 2013 for a review), except for the human gait – an eminent skill for human beings. While the biomechanical and functional structure of the human gait has been studied extensively (see Perry and Burnfield, 2010 for an overview), no studies exist that have investigated the MREP structures underlying the normal human gait and how these correspond to functional components such as spatio-temporal gait parameters. Furthermore, to date there is no evidence whether, or to what extent, functional and biomechanical changes of the human gait, associated with normal aging or musculoskeletal disorders/injuries of the lower extremities, are related to changes in action-related knowledge of the human gait in long-term memory (LTM).

Aging is commonly associated with changes in the biomechanics of gait (Winter et al., 1990; Kerrigan et al., 1998; see McGibbon, 2003 for a review), including a decline in gait speed (Ko et al., 2010; White et al., 2013) from the seventh decade of life (Himann et al., 1988). Gait speed is increasingly recognized as a performance variable and an indicator in the identification of mobility limitations (Bohannon and Glenney, 2014). Studies have shown that people with musculoskeletal injuries of the lower extremities walk slower than healthy people in the long term (McGibbon and Krebs, 2004; Magyar et al., 2012; Gokeler et al., 2013). In community-dwelling older adults, the measurement of gait speed at a comfortable pace has been proven to be a quick, inexpensive and highly reliable measure for evaluating lower extremity function (Guralnik et al., 2000; Shinkai et al., 2000; Verghese et al., 2011) and adverse outcomes, i.e., disability, cognitive impairment, institutionalization, falls and/or mortality (Abellan van Kan et al., 2009; see Peel et al., 2012 for a review). Montero-Odasso et al. (2005) showed that a gait velocity below 0.7 m/s is a reliable predictor of falls. However, prominent independent risk factors for falls in elderly people are not only the impairment of gait, but also cognitive deficits (Montero-Odasso et al., 2012).

Although gait is a largely automated motor task, cognitive resources are required for normal walking (Yogev-Seligmann et al., 2008). The relationship between gait dysfunction and cognitive impairment has been evaluated in various studies (Tabbarah et al., 2002; Atkinson et al., 2007; Allali et al., 2013). Impaired attention and executive functions such as working memory are associated with slower gait and falls (see Yogev-Seligmann et al., 2008; Montero-Odasso et al., 2012 for reviews). Kuo et al. (2007) demonstrated that gait speed partially mediates the association between cognition and disability. Furthermore, a progressive reduction of gait speed was observed up to 12 years before the clinical presentation of cognitive impairment (Buracchio et al., 2010). Thus, comparative evaluations of gait speed might not only be a measurement tool of cognitive impairment, but also of cognitive decline.

Gait patterns can drastically change in the course of normal aging (McGibbon, 2003) and in connection with musculoskeletal disorders or severe injuries of the lower limbs (McGibbon, 2003; Magyar et al., 2012; Gokeler et al., 2013). Thus, knowledge about the cognitive memory structures underlying the human gait would certainly aid in obtaining or regaining normal gait in order to reduce the risk of falls especially in clinical and elderly populations.

A well-documented method that provides psychometric data on the structure and dimensions of the MREP of complex movements in LTM is the structural dimensional analysis of MREP (SDA-M) introduced by Schack (2012). As opposed to other techniques (see Hodges et al., 2007 for an overview) this method assesses MREP structures without asking subjects to explicitly state them. Studies using the SDA-M suggested that MREPs of high-level motor skills are hierarchically organized in LTM similar to knowledge taxonomies suggested for object representation (Hoffmann, 1990). MREPs of complex skills from a variety of sports (e.g., Schack and Mechsner, 2006 [tennis]; Bläsing et al., 2009 [dancing]; Weigelt et al., 2011 [judo]; Frank et al., 2013 [golf]; Lex et al., 2015 [soccer]) were found to be functionally structured in tree-like hierarchies in experts, while less skilled athletes’ possessed less-structured MREPs that are poorly related to functional and biomechanical task demands. In line with that research, a recent study on the acquisition of a golf putt (Frank et al., 2013) demonstrated that skill acquisition is associated with functional adaptations in action-related knowledge in LTM as evidenced by training-induced changes in MREP structures. Moreover, research employing SDA-M in children (Stöckel et al., 2012) and stroke patients (Braun et al., 2007) suggests that cognitive structures of manual actions differ as a function of age and health status. Specifically, Braun et al. (2007) compared MREP structures for drinking out of a cup in 16 persons with stroke and 16 age-matched healthy controls. While they found distinct clustering of actions in a tree-like hierarchy in the healthy controls, representation structures in patients with stroke were less structured with action-related knowledge and ordering appearing to be most affected in patients with more severe symptoms.

That said, while nearly all aspects of the human gait have been examined in the past, to date there are no studies which have investigated the cognitive memory structures underlying the human gait either in young or in older adults. The present study sought to investigate these issues within two experiments.

In a first experiment, it was investigated whether gait-specific LTM structures reflect kinematics and kinematic differences in the human gait. Therefore, spatio-temporal (gait speed, step length, and stride length) and temporophasic (stance time, swing time, load response time, single support time and pre-swing time) gait parameters as well as gait variability (coefficient of variation of gait parameters) at comfortable gait speed (assessed with OptoGait) were compared with gait-specific LTM structures (assessed with SDA-M) in 36 young adults. As gait speed has been established as an important performance measure (Bohannon and Glenney, 2014), subjects were divided into a slow-walking and a fast-walking group (based on a median-split for height-adjusted comfortable gait speed). A difference in LTM structures between slow- and fast-walking adults was expected due to the proven interplay between gait speed and cognition (Montero-Odasso et al., 2012).

In a second experiment, gait parameters and MREPs in healthy older subjects were compared with those of young healthy participants. Twelve young adults from the first experiment with a history of musculoskeletal injuries/disorders were excluded from this analysis as these subjects would probably bias the results. Therefore, we assessed gait parameters and gait-specific LTM structures in 24 healthy older adults and compared them with a group of healthy young adults (*n* = 24) from experiment one. As age-related declines in gait performance occur from the seventh decade of life onward (Himann et al., 1988; Hollman et al., 2011), it was hypothesized that spatio-temporal and temporophasic gait parameters as well as gait variability would not be affected until the age of 60 years. Previous evidence of the relationship between gait dysfunction and cognitive impairment with aging (Yogev-Seligmann et al., 2008; Montero-Odasso et al., 2012) led to the hypothesis that stable gait performance up to 60 years of age is accompanied by unchanged LTM structure (SDA-M).

## Materials and Methods

### Subjects

Prior to participation, written informed consent was obtained from all subjects. The cross-sectional study was conducted according to the Declaration of Helsinki and approved by the Ethical Review Committee of the University of Rostock (A 2013-0150).

#### Experiment 1

Thirty-six young adults volunteered to participate in this study. Participants who suffered from severe, acute gait-affecting injuries or reported to be restricted in their gait were excluded prior to testing. The remaining subjects were divided into slow- (*n* = 18) and fast- (*n* = 18) walking adults by splitting height-adjusted gait speed at the median. Demographic and clinical subject characteristics are provided in **Table 1**.

**Table 1:** Experiment 1 – Demographic and clinical subject characteristics as well as and spatio-temporal and temporophasic gait parameters of slow- and fast-walking young subjects.

	Fast (*n* = 18)	Slow (*n* = 18)	*P*	Cohen’s *d*
Men, *n* (%)	6 (33.3)	9 (50.0)	0.310	–
Age, years, Mean (SD)	28.3 (4.3)	27.8 (2.8)	0.685	–
Weight, kg, Mean (SD)	67.7 (12.7)	73.6 (15.4)	0.224	–
Height, m, Mean (SD)	1.73 (0.08)	1.75 (0.08)	0.315	–
Physical activity, h/week, Mean (SD)	3.3 (3.7)	4.1 (3.7)	0.539	–
History of musculoskeletal injuries/disorders, *n*	3	11	–	–
Cruciate ligament rupture, *n* (%)	0 (0.0)	2 (11.1)	–	–
Torn meniscus, *n* (%)	1 (5.6)	2 (11.1)	–	–
Cartilage damage knee, *n* (%)	0 (0.0)	1 (5.6)	–	–
Ligament elongation knee, *n* (%)	0 (0.0)	1 (5.6)	–	–
Ligament elongation ankle, *n* (%)	0 (0.0)	2 (11.1)	–	–
Chondromalazia patellae, *n* (%)	0 (0.0)	1 (5.6)	–	–
Hip luxation	0 (0.0)	1 (5.6)	–	–
Prolapsed intervertebral disk, *n* (%)	1 (5.6)	1 (5.6)	–	–
Polyarthritis, *n* (%)	1 (5.6)	0 (0.0)	–	–
Total number of steps analyzed	27.67 (3.91)	32.11 (4.76)	0.004^∗∗^	–
Gait speed/height	0.89 (0.07)	0.75 (0.07)	<0.001^∗∗^	2.00
CV_Gait speed_, %	3.41 (1.67)	3.51 (1.44)	0.847	0.06
Step length/height	45.14 (2.71)	39.96 (2.92)	<0.001^∗∗^	1.85
CV_Step length_, %	2.56 (1.42)	3.03 (1.53)	0.346	0.32
Stride length/height	90.41 (5.50)	79.95 (5.79)	<0.001^∗∗^	1.85
CV_Stride length_, %	1.74 (0.72)	2.12 (0.97)	0.183	0.44
Stance time, %GC	60.62 (1.30)	62.31 (1.59)	0.001^∗∗^	1.16
CV_Stance time_, %	2.00 (0.97)	1.86 (0.43)	0.591	0.19
Swing time, %GC	39.35 (1.29)	37.72 (1.61)	0.002^∗∗^	1.12
CV_Swing time_, %	3.16 (1.39)	3.20 (0.91)	0.911	0.03
Load response time, %GC	10.72 (1.29)	12.23 (1.63)	0.004^∗∗^	1.03
CV_Load response time_, %	8.95 (3.23)	8.47 (2.44)	0.614	0.17
Single support time, %GC	39.24 (1.23)	37.79 (1.60)	0.005^∗∗^	1.02
CV_Single support time_, %	2.57 (0.77)	3.13 (0.66)	0.024^∗^	0.78
Pre-swing time, %GC	10.87 (1.22)	12.35 (1.59)	0.004^∗∗^	1.04
CV_Pre-swing time_, %	9.04 (2.94)	8.58 (2.25)	0.602	0.18

*CV, coefficient of variation; %GC, % gait cycle; d, Cohen’s d effect size. Data are presented as means (standard deviation). ^∗^Denotes a significant difference between groups (^∗^P ≤ 0.025, ^∗∗^P ≤ 0.005)*.

#### Experiment 2

Based on the impact of previously incurred musculoskeletal lower limb injuries on gait biomechanics and MREP found in the first experiment, 12 young subjects with a history of lower limb injuries/disorders were excluded. Consequently, 24 healthy young adults from the first experiment were compared to 24 healthy community-dwelling older adults. **Table 2** shows the subjects’ characteristics in the second experiment.

**Table 2:** Experiment 2 – Demographic subject characteristics, spatio-temporal and temporophasic gait parameters of healthy young and elderly subjects.

	Young (*n* = 24)	Elderly (*n* = 24)	*p*	*F*	$\eta_{p}^{2}$	*f*
Men, *n* (%)	10 (41.7)	6 (25.0)	0.221	–	–	–
Age, years, Mean (SD)	27.9 (3.4)	60.3 (6.7)	<0.001^∗∗^	–	–	–
Weight, kg, Mean (SD)	70.4 (15.6)	72.5 (13.8)	0.621	–	–	–
Height, m, Mean (SD)	1.74 (0.08)	1.68 (0.10)	0.009^∗^	–	–	–
Physical activity, h/week, Mean (SD)	3.8 (3.7)	1.4 (2.1)	0.008^∗^	–	–	–
Total number of steps analyzed	29.08 (5.14)	30.63 (3.84)	0.245	–	–	–
CV_Gait speed_, %	3.47 (1.40)	2.82 (1.40)	0.124	2.454	0.052	0.234
Step length/height	43.19 (3.67)	43.19 (3.67)	0.995	<0.001	<0.001	<0.032
CV_Step length_, %	2.66 (1.17)	2.65 (1.17)	0.975	0.001	<0.001	<0.032
Stride length/height	86.50 (7.36)	86.41 (7.36)	0.953	0.003	<0.001	<0.032
CV_Stride length_, %	1.85 (0.74)	1.83 (0.74)	0.926	0.009	<0.001	<0.032
Stance time, %GC	61.08 (1.98)	60.41 (1.98)	0.261	1.294	0.028	0.170
CV_Stance time_, %	1.89 (0.75)	1.98 (0.75)	0.707	0.143	0.003	0.055
Swing time, %GC	38.93 (1.98)	39.60 (1.98)	0.265	1.276	0.028	0.170
CV_Swing time_, %	3.10 (1.14)	3.01 (1.14)	0.951	0.004	0.001	0.032
Load response time, %GC	11.10 (1.99)	10.34 (1.99)	0.212	1.603	0.034	0.188
CV_Load response time_, %	8.43 (3.48)	11.05 (3.48)	0.016^∗^	6.304	0.123	0.375
Single support time, %GC	38.87 (1.94)	39.64 (1.94)	0.194	1.740	0.037	0.196
CV_Single support time_, %	2.69 (0.85)	3.46 (0.85)	0.005^∗∗^	8.943	0.166	0.446
Pre-swing time, %GC	11.26 (1.92)	10.44 (1.92)	0.158	2.059	0.044	0.215
CV_Pre-swing time_, %	8.42 (4.43)	11.70 (4.43)	0.017^∗^	6.098	0.119	0.368

*CV, coefficient of variation; %GC, % gait cycle; f, Cohen’s f effect size. Data are presented as mean (standard deviation). ^∗^Denotes a significant difference between groups (^∗^P ≤ 0.025, ^∗∗^P ≤ 0.005)*.

### Experimental Procedure

The subjects participated in one experimental session that included the measurement of gait parameters and MREP.

#### Gait Analysis

Spatio-temporal gait parameters were assessed using the OptoGait photoelectric cell system (Microgate, Bolzano, Italy) in a quiet room, with no auditory or visual interference for the subjects. Six transmitting and six receiving bars were placed parallel to each other (distance between bars: 1 m). The subjects were instructed to wear closed shoes with heel height not exceeding 3 cm (Kressig and Beauchet, 2006). They walked along a 6-m walkway at a self-selected comfortable speed starting and stopping each walk 2 m before and after the walkway. Subjects performed one familiarization and five experimental trials. The rest interval between the tests was 1 min. Data were sampled at 1 kHz and analyzed using the OptoGait software (version 1.8.0.0., Microgate, Bolzano, Italy) and Excel 2007 (Microsoft Inc., Seattle, WA, USA). We collected data from three spatio-temporal (gait speed, step length, stride length) and five temporophasic (stance time, swing time, loading response time, single support time and pre-swing time in percentage of the gait cycle) parameters. Spatio-temporal parameters were adjusted to height. Moreover, gait variability [coefficient of variation [CV] = (standard deviation [SD]/mean [M]) x 100] was calculated for all gait parameters. Gait variability, especially stride-to-stride variability, is associated with falls in elderly subjects (Hausdorff, 2007). The mean value of all steps was used for data analysis. The OptoGait system demonstrated high validity and reliability for the assessment of gait parameters compared with a validated electronic walkway (GAITRite) in young and older subjects as well as in orthopedic patients (Lienhard et al., 2013; Lee et al., 2014).

#### Structural Dimensional Analysis of Mental Representation

**FIGURE 1:**
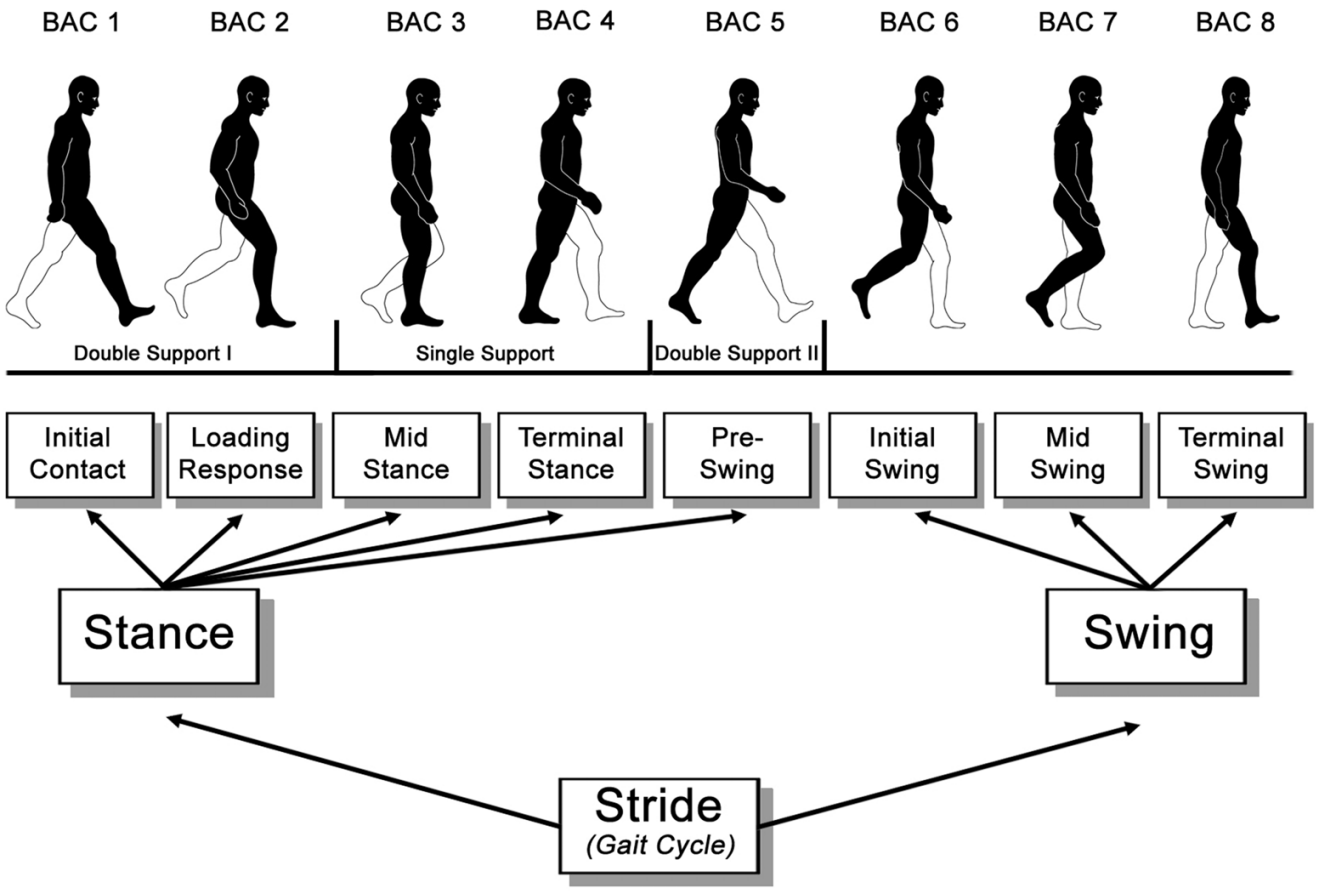
**Functional divisions of the gait cycle according to Perry and Burnfield (2010)**.

With regard to generally recognized models of the human gait (Perry and Burnfield, 2010), a gait cycle consists of two main phases – the stance and swing phase – and eight functional periods – initial contact, loading response, mid stance, terminal stance, pre-swing, initial swing, mid swing, and terminal swing (see **Figure 1**).

**FIGURE 2:**
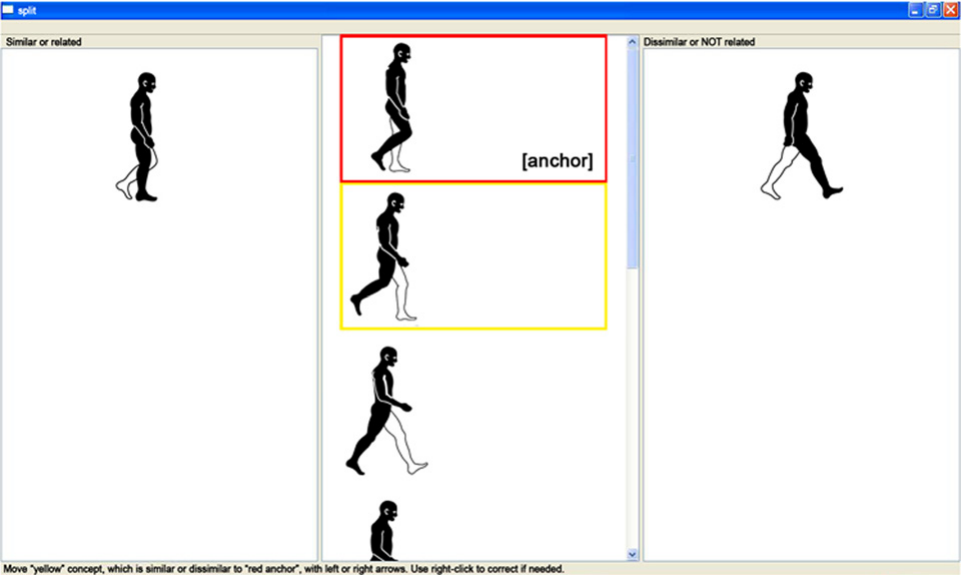
**Screenshot of the splitting procedure.** One picture was presented in the anchoring position (top picture in the middle column as indicated by a red rectangle) to which participants classified the remaining N-1 pictures (presented in the lower half of the middle comlumn as indicated by a yellow rectangle) as similar (left column) or dissimilar (right column) to the anchoring picture.

Pictures of seven functional periods, excluding terminal swing (see **Figure 1**), were used as basic action concepts (BACs; i.e., key conceptual structures of a movement within the memory system) of the human gait in the splitting procedure of the SDA-M. The last period, terminal swing, was not used for the splitting procedure as pilot data demonstrated that terminal swing and initial contact are not clearly distinguishable from each other and, thus, would distort the results. During the splitting procedure, the pictures were presented on a 19 inch computer screen with always one picture in the anchoring position (i.e., a reference picture located at the top of the screen) to which participants classified the remaining N-1 pictures (presented in the lower half of the computer screen) as similar or dissimilar to the anchoring picture (see **Figure 2**). Participants were asked to decide via pressing keys for ‘yes’ and ‘no’ whether the presented pictures are closely related in the gait cycle or not. After all judgments were made for an anchoring picture, another BAC randomly occupied the anchor position, and all other BACs were compared to this anchoring picture until each of the BACs was in the anchoring position. This splitting procedure delivered an Euclidean distance scaling between all BACs of the gait cycle. A following hierarchical cluster analysis then outlined the individual structure of the given set of BACs with a factor analysis revealing the feature dimensions of these individual cluster solutions (for more detail regarding SDA-M see Schack, 2012 and supplementary material to Stöckel et al., 2012).

### Statistical Analysis

#### Spatio-Temporal and Temporophasic Gait Parameters

Data were checked for normal distribution using the Kolmogorov–Smirnov test. Differences between the groups (Experiment 1: slow vs. fast; Experiment 2: young vs. older) were tested for significance with the chi-squared tests, unpaired Student’s *t*-tests or analyses of covariance (ANCOVA) adjusted for physical activity. The level of significance was set at *P* ≤ 0.025 (alpha-adjustment for conducting two tests [first and second experiment] *P* ≤ 0.050/2 = 0.025). All data were analyzed using the SPSS statistical package 20.0 (SPSS Inc., Chicago, IL, USA). The effect size was calculated with G^∗^Power (version 3.1.4.; Faul et al., 2007). The effect sizes *d* and *f* were interpreted using the classification of Cohen (1988): *d* = 0.20 small effect, *d* = 0.50 moderate effect, *d* = 0.80 large effect, *f* = 0.10 small effect, *f* = 0.25 moderate effect, *f* = 0.40 large effect. Data are presented as M (SD) in the tables. If appropriate, data are reported as mean difference (MD) and 95% confidence interval (95% CI).

#### Mental Representation of the Gait Structure

For all cluster analyses a critical value of *d*_crit_ = 3.53, reflecting an alpha level of α = 0.050, was used to determine the statistical relevance of links between BACs with only links below this critical value being considered as statistically relevant (i.e., related to each other). Between-group comparisons (Experiment 1: slow vs. fast; Experiment 2: young vs. older) of the cluster solutions derived from SDA-M were performed by determining the structural invariance (λ) between the grouped cluster solutions (i.e., mean group dendograms derived from cluster analysis by summing the individual Z-matrices). According to Schack (2012), an invariance measure of λ < λ_crit_ = 0.68 was used to determine significance at an alpha level of α = 0.050. Moreover, we captured the number of sequencing errors (i.e., incorrect links between single BACs based on the predefined order of BACs, **Figure 1**) from the individual cluster solutions and compared the average values between the groups using the unpaired Student’s *t*-test.

## Results and Discussion

### Experiment 1

Slow- and fast-walking young adults did not differ significantly in anthropometric data and physical activity.

#### Spatio-Temporal and Temporophasic Gait Parameters

A total number of 32 and 28 steps was analyzed in slow- and fast-walking young adults, respectively (**Table 1**). The mean values of self-selected walking speeds were as follows: 1.32 m/s (±0.14 m/s) for the slow-walking group; 1.53 m/s (±1.52 m/s) for the fast-walking group.

The difference in gait speed/height between fast- and slow-walking adults was significant (15.7%; MD = –0.14; 95% CI: –0.18 to –0.09). The two spatio-temporal gait parameters (step length/height, stride length/height) were reduced in the slow-walking group by 11.5% (MD = –5.18; 95% CI: –7.09 to –3.28) and 11.6% (MD = –10.46; 95% CI: –14.29 to –6.64), respectively. Slow-walking adults further demonstrated a significantly longer stance time (2.8%; MD = 1.69%; 95% CI: 0.70 to 2.67%) and shorter swing time (–4.5%; MD = –1.63%; 95% CI: –2.62 to –0.64%). Within the stance phase, single support time was shorter (–3.7%; MD = –1.45%; 95% CI: –2.42 to –0.48%) and load response time (14.1%; MD = 1.51%; 95% CI: 0.52 to 2.51%) as well as pre-swing time (13.6%; MD = 1.48%; 95% CI: 0.52 to 2.44) were longer in the slow-walking group. The only difference in CV could be documented for single support time with a higher value (21.8%; MD = 0.56%; 95% CI: 0.08 to 1.0%) in slow-walking adults. Spatio-temporal and temporophasic gait parameters and corresponding CV are reported in **Table 1**.

#### Mental Representation of the Gait Structure

**FIGURE 3:**
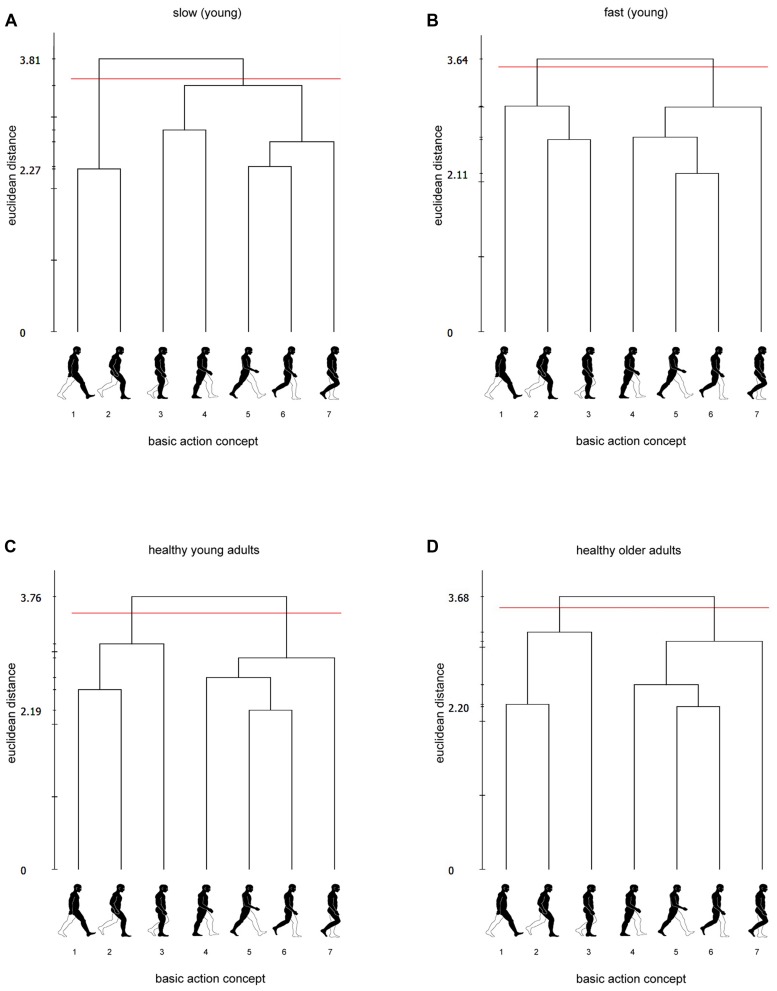
**Mean group dendograms for (A) slow-walking adults, (B) fast-walking adults, (C) healthy young adults and (D) healthy older adults.** The y-axis displays the euclidean distance. The horizontal line marks d_crit_ for a given α-level (d_crit_ = 3.53; α = 0.050). The x-axis shows the BACs (basic action concepts): (1) initial contact, (2) loading response, (3) mid stance, (4) terminal stance, (5) pre-swing, (6) initial swing, (7) mid swing.

As can be seen in **Figures 3A,B**, cluster analysis of mean group dendograms revealed distinct clustering (with critical value *d*_crit_ = 3.53) in both slow- and fast-walking young adults. In both groups BACs have been clustered in two functional units, but of different structures. In slow-walking adults there were distinct clusters for BACs 1–2 (initial contact, loading response) and BACs 3–7 (mid stance, terminal stance, pre-swing, initial swing, mid swing; *d* = 3.81, *P* < 0.050) while in fast-walking adults there were two distinct clusters for BACs 1-3 and BACs 4–7 (*d* = 3.64, *P* < 0.050). Statistical analysis of invariance revealed the structural differences between slow- and fast-walking adults to be significant (λ = 0.51, *P* < 0.050). The number of sequencing errors did not differ between slow- (*M* = 0.50, SD = 0.62) and fast-walking adults (*M* = 0.39, SD = 0.61; *P* = 0.590).

#### Discussion

In the first experiment, we evaluated whether gait-specific LTM structures reflect kinematic differences in the human gait. The established relationship between gait speed and executive function such as working memory (Montero-Odasso et al., 2012) led to the hypothesis that gait performance and MREP differ between slow- and fast-walking young adults. The present results confirm this assumption. Bohannon (1997) established reference values for the comfortable gait speed of adults 20–79 years of age and showed that height-normalized gait speed ranged from 0.83 for men to 0.86 for women in their thirties. Consequently, the height-normalized gait speed of the slow-walking group (0.75) is well below these normative values indicating reduced gait performance. This lower comfortable gait speed was associated with differences in spatio-temporal and temporophasic parameters, i.e., shorter step and stride lengths, longer stance and double support times as well as shorter swing and single support times. Furthermore, these differences in gait performance associated with lower comfortable gait speeds are reflected by structural differences in gait-related memory structures in LTM.

Cluster analysis revealed two functional clusters for each group with BAC 3 (mid stance) clustered together with BACs 1–2 (initial contact, loading response) in the fast-walking group or with BACs 4–7 (terminal stance, pre-swing, initial swing, mid swing) in the slow-walking group, suggesting that the mid stance (i.e., first part of the single limb support phase) is a critical functional period for gait speed. The separation of the two functional periods within the single limb support phase (BACs 3–4) in the fast-walking adults suggests that for a well-coordinated gait cycle the single limb support phase serves as a (critical) transition between the two double support phases (and the changing responsibilities of the two feet). In slow-walking adults this transition seems to be impaired (reduced single limb support times along with a separate clustering of the first double and single limb support phases) resulting in prolonged double support phases and a reduced gait speed which are probably due to a history of musculoskeletal injuries. We have documented a total of 11 previously incurred musculoskeletal injuries for the slow walkers (13–144 months before) but only two injuries and one disorder for fast walkers (48–132 months before; **Table 1**). In the slow-walking group, 64% of the participants suffered from knee injuries.

It has been shown that subjects with knee injuries may have altered gait performance, i.e., walk slower than healthy people (Gao and Zheng, 2010; Gokeler et al., 2013 [anterior cruciate ligament deficiency or reconstruction]; Magyar et al., 2012 [meniscus injury, meniscectomy]). Perry and Burnfield (2010) divided the pathological mechanisms for disease-related walking abnormalities into five functional categories, i.e., deformity, muscle weakness, sensory loss, pain and impaired motor control. Each subject has an individual mixture of impairments which alters his walking ability. These altered gait patterns may persist for years as shown in a review by Gokeler et al. (2013) for patients with anterior cruciate ligament reconstruction. These findings correspond with the present results. Although none of the participants reported current discomfort or pain either during daily walking or while attending the tests, walking abnormalities continue to exist over the long term, whereby the results of SDA-M indicate that the single limb support phase may serve as a critical functional period for gait speed. As during the single limb support phase, and in particular during mid stance, the total responsibility for supporting body weight is on one leg, limb stability is a major objective during this phase. A previous study of subjects with anterior cruciate ligament deficiencies found that knee instability is compensated by a stiffening strategy involving higher muscle activity and lower knee motion during mid stance (Hurd and Snyder-Mackler, 2007).

Thus, the present results indicate that people with previously incurred musculoskeletal injuries of the lower extremities still exhibit changes in gait biomechanics sustainably affecting cognitive structures of the gait.

### Experiment 2

With regard to the differences between slow- and fast-walking adults found in experiment one, 12 young adults from the first experiment with a history of musculoskeletal injuries/disorders were excluded from the following analyses. Healthy young and elderly adults differed in height and physical activity. Elderly subjects were significantly smaller and less physically active than young adults (**Table 2**). Consequently, all spatio-temporal gait parameters were normalized to height and physical activity was entered as a covariate into an ANCOVA to account for these differences between groups.

#### Spatio-Temporal and Temporophasic Gait Parameters

A total number of 29 steps was analyzed in young adults and 31 steps in elderly adults (**Table 2**). The mean values of self-selected walking speeds did not differ significantly between groups (*F* = 0.022; *P* = 0.715; $\eta_{p}^{2}$ = <0.001) and were as follows: 1.46 m/s (±0.16 m/s) for the young group; 1.45 m/s (±0.16 m/s) for the elderly group (MD = 0.01 m/s; 95% CI: –0.09 to 0.10).

No significant differences in spatio-temporal or temporophasic parameters were documented. However, the CV of load response time, single support time and pre-swing time were significantly lower by 23.7% (MD = –2.62%; 95% CI: –4.72 to –0.52%), 22.3% (MD = –0.76%; 95% CI: –1.28 to –0.25%) and 28.2% (MD = –3.28%; 95% CI: –5.95 to –0.60%) in young subjects, respectively. Further between-group differences in CV were not observed. **Table 2** shows the *M* and SD of spatio-temporal and temporophasic gait parameters and corresponding CVs.

#### Mental Representation of the Gait Structure

Gait-specific representation structures of healthy young and older adults are displayed in **Figures 3C,D**. Cluster analysis of mean group dendograms revealed that in both healthy young (*d* = 3.76, *P* < 0.050) and healthy older adults (*d* = 3.68, *P* < 0.050) BACs have been clustered in two functional units forming distinct clusters for BACs 1-3 (initial contact, loading response, mid stance) and BACs 4-7 (terminal stance, pre-swing, initial swing, mid swing). Statistical analysis of invariance revealed the cluster solutions to be identical (λ = 1.0, *P* = 1.0). The number of sequencing errors was higher in older (*M* = 0.92, SD = 1.00) than in young adults (*M* = 0.44, SD = 0.61; *P* = 0.020).

#### Discussion

In the second experiment, healthy young and elderly subjects were compared with respect to gait performance and MREPs. Himann et al. (1988) have shown that gait speed decreases by 12–16% per decade after the age of 63 years. Furthermore, Hollman et al. (2011) even reported a decrease past the age of 80. Thus, it was hypothesized that gait parameters are stable until the age of 60 accompanied by unchanged MREPs. Our current results confirm these previous findings. Normal aging led to a higher variability of loading response time, single support time and pre-swing time, but no changes of the spatio-temporal and temporophasic gait parameters or the underlying gait-related memory structures in LTM. According to normative criteria established by Studenski (2009), comfortable absolute gait speed was superior in the elderly subjects. Bohannon (1997) defined reference values for height-normalized gait speed for adults 60 years of age and revealed a range of 0.78 for men to 0.80 for women. Thus, the height-normalized gait speed of the older adults of the present study (0.87) was slightly above these normative values, indicating excellent performance. The present results indicate that the human gait and underlying gait-related memory structures in LTM are stable until the age of 60 years, thereby providing support for the validity of the SDA-M to assess gait-related memory structures.

Although the higher number of sequencing errors is not reflected by the average representation structure, it is probably associated with the higher CV in older adults.

## Conclusion

Spatio-temporal and temporophasic parameters of gait at a comfortable gait speed differed between slow- and fast-walking young adults. These differences in human gait patterns associated with lower comfortable gait speeds are reflected by structural differences in gait-related memory structures in LTM, probably due to previously incurred musculoskeletal injuries. The results further indicate that the human gait and its MREP are stable until the age of 60 in healthy subjects.

Notably, LTM representations of the gait were found to be functionally structured in tree-like hierarchies across all participants in the present study. As well-structured, hierarchically organized mental representations are typically associated with expert performance (see Land et al., 2013 for a review), this finding indicates that gait is a high-level motor skill in all populations tested within the present study. That said, considering cluster solutions of healthy adults to be representative of normal or optimal gait-related memory structures, it appears that the biomechanical structure of the human gait (see Perry and Burnfield, 2010) is not clearly reflected by the gait-related LTM structure. In contrast to separating stance and swing phases (Perry and Burnfield, 2010), healthy adults formed two distinct clusters comprised of BACs 1–3 and BACs 4–7 (**Figure 1**). Thereby, the single limb support phase (BACs 3–4) seems to be an important transition phase for a well-coordinated gait performance which is not strongly reflected in LTM structure and clustered to the preceding and subsequent phases. The results indicate that MREP of the human gait rather draws on an optimal interaction between both feet and the changing responsibilities than on functional phases within one foot.

With regard to a potential clinical use of the SDA-M, future research should examine how gait-related memory structures are affected by different disorders of the lower extremities, how long structural abnormalities persist in LTM and which therapeutic measures are most efficient for regaining normal gait patterns. Moreover, normative cluster solutions of human gait patterns for various age groups, including smaller age spans in children and older adults (e.g., in steps of 5 years in older adults from 60 years on, and probably even smaller steps in children until the age of 20), should be developed to inform and support clinicians regarding the diagnosis and monitoring of gait abnormalities based on cognitive structures.

Notwithstanding the necessity of extended research on this topic, SDA-M may be considered as a valuable clinical tool for the diagnosis of (current and previous) gait abnormalities and for monitoring the effectiveness of therapeutic measures.

## Author Contributions

TS, AM-M, and MB developed the study concept, and all of these authors contributed to the design in collaboration with RS and RB. RJ, MB, and AM-M collected the data and analyzed it in collaboration with TS. AM-M and TS wrote the first draft of the manuscript, and MB, RJ, RS, and RB helped to edit and revise it. All authors approved the final, submitted version of the manuscript.

## Conflict of Interest Statement

The authors declare that the research was conducted in the absence of any commercial or financial relationships that could be construed as a potential conflict of interest.
